# Tertiary Lymphoid Structures as Independent Predictors of Favorable Prognosis in Muscle‐Invasive Bladder Cancer

**DOI:** 10.1002/cam4.70978

**Published:** 2025-05-21

**Authors:** Xiaodong Teng, Zhen Chen, Yanfeng Bai, Hui Cao, Jing Zhang, Liming Xu, Kaihua Liu, Yuqian Shi, Yang Shao

**Affiliations:** ^1^ Department of Pathology, the First Affiliated Hospital Zhejiang University School of Medicine Hangzhou China; ^2^ Nanjing Geneseeq Technology Inc. Nanjing Jiangsu China

**Keywords:** tertiary lymphoid structures, TP53 genes, tumor microenvironment, urinary bladder neoplasms

## Abstract

**Background:**

Tertiary lymphoid structure (TLS) has been reported to be associated with prognosis and immunotherapy in certain cancers. The objective of our study was to investigate the prognostic significance of Tertiary Lymphoid Structures (TLS) within the context of Muscle‐Invasive Bladder Cancer (MIBC), while concurrently examining the clinicopathological and molecular determinants influencing TLS formation.

**Methods:**

Immunohistochemistry was used to detect the expression of TLS, CD8+ T cells, B cells, and plasma cells in 119 MIBC cases, of which 80 cases were tested by next generation sequencing (NGS) for analyzing the differences in gene alterations between TLS‐negative and TLS‐positive.

**Results:**

TLS were identified in 52.1% (62/119) of the MIBC cases studied. Patients exhibiting TLS demonstrated reduced T and TNM staging and prolonged overall survival (OS) compared to those lacking TLS. Multivariate analysis showed that TLS was an independent prognostic factor. Densities of B cells, CD8+ T cells, and plasma cells in tumors were significantly correlated with TLS, but in the cases with low‐density B cells, high‐density CD8+ T cells, or high‐density plasma cells, differences in OS between the tumors with TLS and without TLS were not significant. Compared with TLS‐negative tumors, TLS‐positive tumors had a lower frequency of *TP53* mutations and higher frequencies of *FAT1* and *CDKN1A* mutations. Tumor mutational burden (TMB) was not significantly different between the two groups but was significantly associated with TLS in *TP53* wild‐type tumors.

**Conclusions:**

TLS emerged as an independent harbinger of favorable prognosis in MIBC, predominantly mediating antitumor responses via B cells. Moreover, *TP53* mutations were identified as a potential inhibitor of TLS formation.

## Graphical abstract

Tertiary lymphoid structure (TLS) was an independent predictor of favorable prognosis in MIBC, and mainly played antitumor role effect through B cells. Through NGS analysis of tumor samples, we also believe that TP53 mutations could inhibit the formation of TLS.

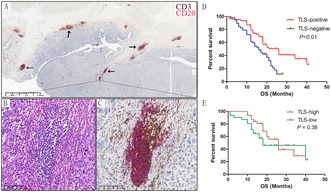

## 1 Background

Bladder cancer, with its invasive muscle phenotype, stands as a formidable challenge in oncology due to its aggressive nature and the limited efficacy of current treatment options. Muscle‐invasive bladder cancer (MIBC) marks a critical stage where the tumor's metastatic potential sharply escalates, demanding the identification of novel prognostic markers and therapeutic targets [1].

Within the complex landscape of the tumor microenvironment (TME), the presence of tertiary lymphoid structures (TLS) has garnered significant attention. These immune cell clusters resemble lymphoid tissues and are found within the supportive stroma of diverse solid tumors. They are believed to regulate local immune responses, potentially shaping the course of cancer progression and the effectiveness of immunotherapeutic approaches [2, 3, 4, 5].

Emerging evidence suggests that the presence of TLS is associated with improved prognosis and is more frequently observed in MIBC than in non‐muscle‐invasive forms of the disease [5, 6, 7]. This has led to the hypothesis that TLS may have a correlation with tumor aggressiveness. Such findings have ignited further inquiry into the mechanisms influencing the clinical trajectory of MIBC and the potential of TLS as prognostic biomarkers.

By undertaking a thorough examination of TLS in MIBC, we further advance this field. To this end, we aim to delineate the correlation between TLS and clinical outcomes, thus providing a more nuanced understanding of their prognostic importance and their interplay with the clinicopathological and molecular aspects of MIBC. This investigation is poised to enhance our understanding of the role of TLS in MIBC.

## 2 Methods

### 2.1 Patients

The study cohort comprised 119 patients diagnosed with MIBC and treated with radical cystectomy at the First Affiliated Hospital of Zhejiang University School of Medicine from 2016 to 2018. All cases were re‐evaluated by two experienced pathologists (Y.B and X.T) to confirm treatment naïveté at the time of study entry. Twenty representative 4 μm‐thick sections from formalin‐fixed paraffin‐embedded (FFPE) tumor blocks were selected for subsequent immunohistochemical and next‐generation sequencing (NGS) analyses. Tumor staging adhered to the Eighth Edition of the Union for International Cancer Control/American Joint Committee on Cancer guidelines, with patient survival data collected through December 2019. The peripheral blood neutrophil and lymphocyte counts, obtained from clinical records within 2 weeks post‐surgery, were used to calculate the Neutrophil‐to‐Lymphocyte Ratio (NLR), with the high and low NLR groups defined by the median value of 2.82 from all measurements. The study protocol was approved by the hospital's Ethics Committee and aligned with the Declaration of Helsinki principles.

### 2.2 TLS Evaluation

The presence and maturity of TLS were evaluated in a blinded manner by two pathologists using CD3‐CD20 immunohistochemical staining, with discrepancies resolved by consensus. TLS was defined as aggregates exceeding 50 B and T lymphocytes, with mature TLS (mTLS) distinguished by the presence of germinal centers on hematoxylin and eosin‐stained sections, as previously described [8]. TLS density was calculated as the number of TLS per tumor area using a slice scanning system (Konfoong Biotech, Ningbo, China). Samples were categorized into TLS‐high or TLS‐low based on their density relative to the median value of the TLS‐positive group.

### 2.3 Immunohistochemistry Staining

The double CD3‐CD20 immunohistochemistry staining was performed with the primary antibodies CD3 and CD20 (all antibody information can be found in Table S1). The staining procedure was performed with a Bond‐III Automated IHC Staining System (Leica, Germany) following the manufacturer's recommendations.

CD8 and CD138 staining were performed with the Bond‐III Automated IHC Staining System with Bond Polymer Refine detection kits (Leica, Germany), also according to the manufacturer's instructions. Slides were scanned with the PANNORAMIC 250 Flash III DX system (3DHISTECH, Hungary). CD8+ T cell densities were evaluated by pathologists (Y.B) using the QuanCenter automated analysis system (3DHISTECH, Hungary). Plasma cell counts were determined in five high‐power fields on CD138‐stained sections, while B cell counts were assessed in 10 high‐power fields on CD3‐CD20‐stained sections, with only CD20‐stained lymphocytes being scored. Density categorization was based on median cell count. Plasma cell counts were enumerated in five immune cell‐positive hot 20 × fields on CD138‐stained sections. B cell counts were enumerated in 10 immune cell‐positive hot 20 × fields on CD3‐CD20‐stained sections (only the lymphocytes stained with CD20 were scored). Densities were stratified using median cell count as the cutoff.

PD‐L1 staining was conducted according to the manufacturer's guidelines for the Ventana Medical Systems (Tucson, AZ, USA). Expression levels were evaluated using the tumor proportion score (TPS), immune cell proportion score (IPS), and combined positive score (CPS) as described by Shitara et al. [9]. Positive PD‐L1 scores were defined as 25% or higher.

### 2.4 Next‐Generation Sequencing (NGS)

NGS was performed on 80 samples, with genomic DNA extracted using the QIAamp DNA FFPE Tissue Kit (Qiagen, Germany). DNA quality was assessed via 1% agarose gel electrophoresis, and degraded samples were excluded. Quantification was achieved using a Qubit 3.0 fluorometer and the Qubit dsDNA HS assay kit (Life Technologies, USA). Library preparation for hybrid‐based targeted NGS utilized the GeneseeqPrime Kit (Geneseeq, Nanjing, China), covering 425 cancer‐associated genes listed in Table S2. Sequencing was conducted on the Illumina Nextseq550 platform, with data processed using the manufacturer's bioinformatics software. Tumor mutational burden (TMB) and microsatellite instability (MSI) were also assessed.

### 2.5 Statistical Analysis

Overall survival (OS) was measured from the date of diagnosis to the date of death or last follow‐up. Survival analysis was conducted using the Kaplan–Meier method with log‐rank testing and Cox regression. Associations between groups and clinicopathological characteristics were evaluated using chi‐square or Fisher's exact tests, and nonparametric correlations assessed using the Mann–Whitney test. All statistical tests were two‐sided, with *p* < 0.05 considered significant. Analyses were performed using IBM SPSS 28.0 software (Chicago, USA). R packages ‘maftools’ and ‘complexheatmap’ were employed to analyze gene mutation differences between TLS‐positive and TLS‐negative cases.

## 3 Results

### 3.1 TLS Was a Predictor of Good Prognosis in MIBC

TLSs were identified in 52.1% (62/119) of MIBC cases, predominantly at the tumor periphery, with mature TLS (mTLS) observed in only six cases. The prevalence of TLS showed no association with gender, age, N‐stage, vascular invasion, nerve invasion, or PD‐L1 expression. However, a higher incidence of TLS prevalence was observed in cases with lower T‐stage and TNM stage (Table 1).

**TABLE 1:** Associations between TLS and investigative factors.

Factor	TLS‐negative	TLS‐positive	*p*
Gender			
Female	5	11	
Male	52	51	0.152
Age			
< 70 year	23	34	
≥ 70 year	34	28	0.114
Tumor size			
< 3.8 cm	27	35	
≥ 3.8 cm	30	27	0.322
PD‐L1 (TPS)			
Negative	44	45	
Positive	13	17	0.563
PD‐L1 (IPS)			
Negative	51	48	
Positive	6	14	0.079
PD‐L1 (CPS)			
Negative	40	39	
Positive	17	23	0.402
TNM‐stage			
II	7	21	
III	48	39	
IV	2	2	0.018
T‐stage			
2	9	24	
3	34	30	
4	14	8	0.014
N‐stage			
0	43	48	
1	5	5	
2	9	9	0.968
Vascular invasion			
Without	33	41	
With	24	21	0.355
Nerve invasion			
Without	42	52	
With	15	10	0.173
NLR			
Low	26	33	
High	31	29	0.407

Abbreviations: NLR, neutrophil‐to‐lymphocyte ratio; TLS, tertiary lymphoid structure.

**FIGURE 1:**
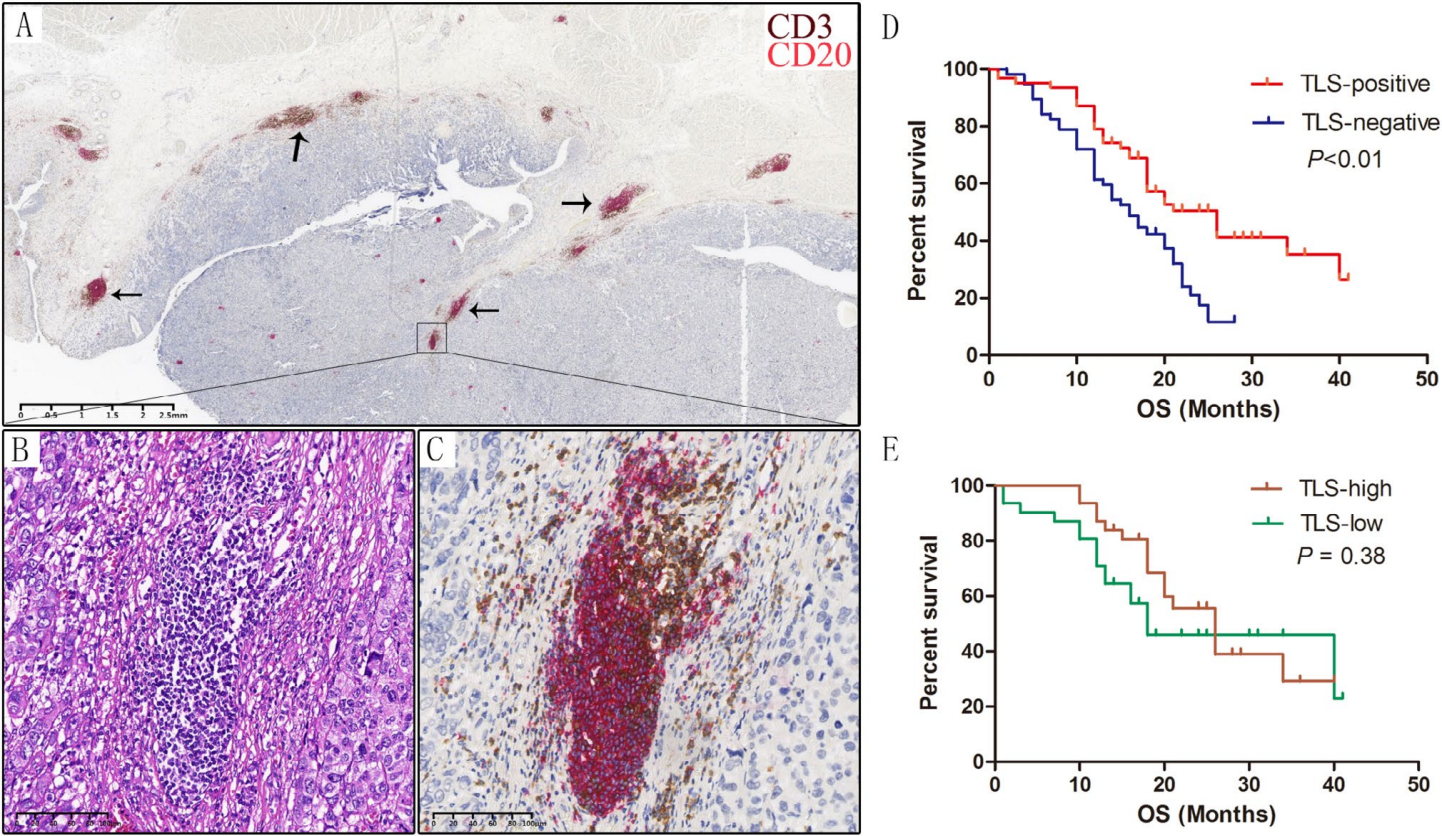
TLS in MIBC. TLS was mainly formed by the aggregation of B cells and T cells (B, C), and most of them are located at the edge of the tumor (A). The OS of patients in the TLS‐positive group was significantly longer than that in the TLS‐negative group (D), and the OS of patients in TLS‐high group was longer than that in TLS‐low group, but not significantly (E).

Log‐rank testing revealed that TLS status, patient age at diagnosis, CD8+ T cell density, and nerve invasion were significant predictors of overall survival (OS) in MIBC patients (Table 2, Table S3). Longer OS was exhibited in the TLS‐positive group compared to the TLS‐negative group (Figure 1D). Although patients with high TLS density (TLS‐high) demonstrated a trend toward longer OS than those with low TLS density (TLS‐low), this trend was not statistically significant (Figure 1E). In addition to the evaluation of TLS status and other clinicopathological factors in relation to OS, a comprehensive evaluation was performed. Cox regression analysis identified TLS status (hazard ratio [HR] 1.701, *p* < 0.05) and patient age at diagnosis (HR 0.556, *p* < 0.05) as independent prognostic factors for OS (Table 2).

**TABLE 2:** Univariate and multivariate analysis of overall survival in MIBC.

Variables	Univariate	Multivariate	
	*p*	HR (95% CI)	*p*
TLS (negative vs. positive)	0.001	1.701 (1.018–2.842)	0.043
Age (< 70 year vs. ≥ 70 year)	0.001	0.556 (0.341–0.9070	0.019
CD8+ cell density (low vs. high)	0.029	1.214 (0.744–1.982)	0.438
Nerve invasion (without vs. with)	0.009	0.641 (0.379–1.087)	0.099

Abbreviations: MIBC, muscle‐invasive bladder cancer; TLS, tertiary lymphoid structure.

### 3.2 TLS Primarily Exerts Anti‐Tumor Effects via B Cells

**FIGURE 2:**
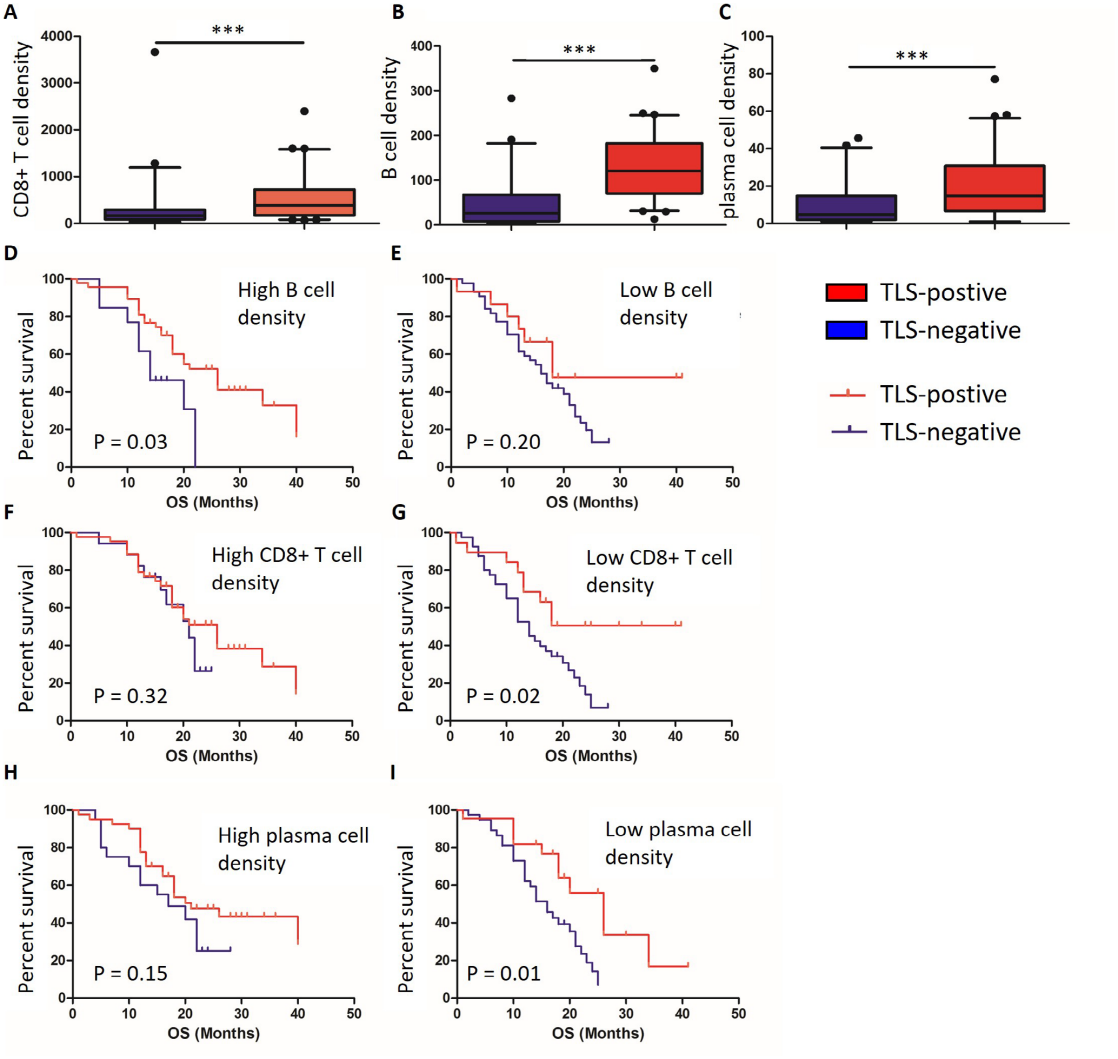
The relationship between TLS and tumor‐infiltrating lymphocytes in MIBC. The densities of CD8+ T cells (A), B cells (B) and plasma cells (C) in the TLS‐positive group were significantly higher than that in the TLS‐negative group. In the cases with high densities of B cells (D), low densities of CD8+ T cells (G) or low densities of plasma cells (I), TLS‐positive patients had longer OS than TLS‐negative patients. However, there was no significant differences in OS between TLS‐positive and TLS‐negative groups in the cases with low‐density B cells (E), high‐density CD8+ T cells (F) or high‐density plasma cells (H). *, *p* < 0.05; **, *p* < 0.01; ***, *p* < 0.001.

To ascertain the primary effector cells involved in TLS, several crucial tumor‐infiltrating lymphocytes (TILs) within the samples, including CD8+ T cells, B cells, and plasma cells, were evaluated. A significant correlation was observed between TLS and the density of these TILs (Figure 2A–C). The TILs were subsequently stratified into high‐ and low‐density groups to assess the prognostic relevance of TLS within each stratum. Notably, in the subgroup with high‐density B cells, TLS were found to be significantly associated with improved OS. In contrast, no substantial correlation was identified between TLS and OS in the high‐density CD8+ T cell and plasma cell subgroups (Figure 2D–I), indicating a predominant role for B cells in mediating the anti‐tumor effects of TLS.

### 3.3 Correlation Between TLS and Molecular Characteristics

**FIGURE 3:**
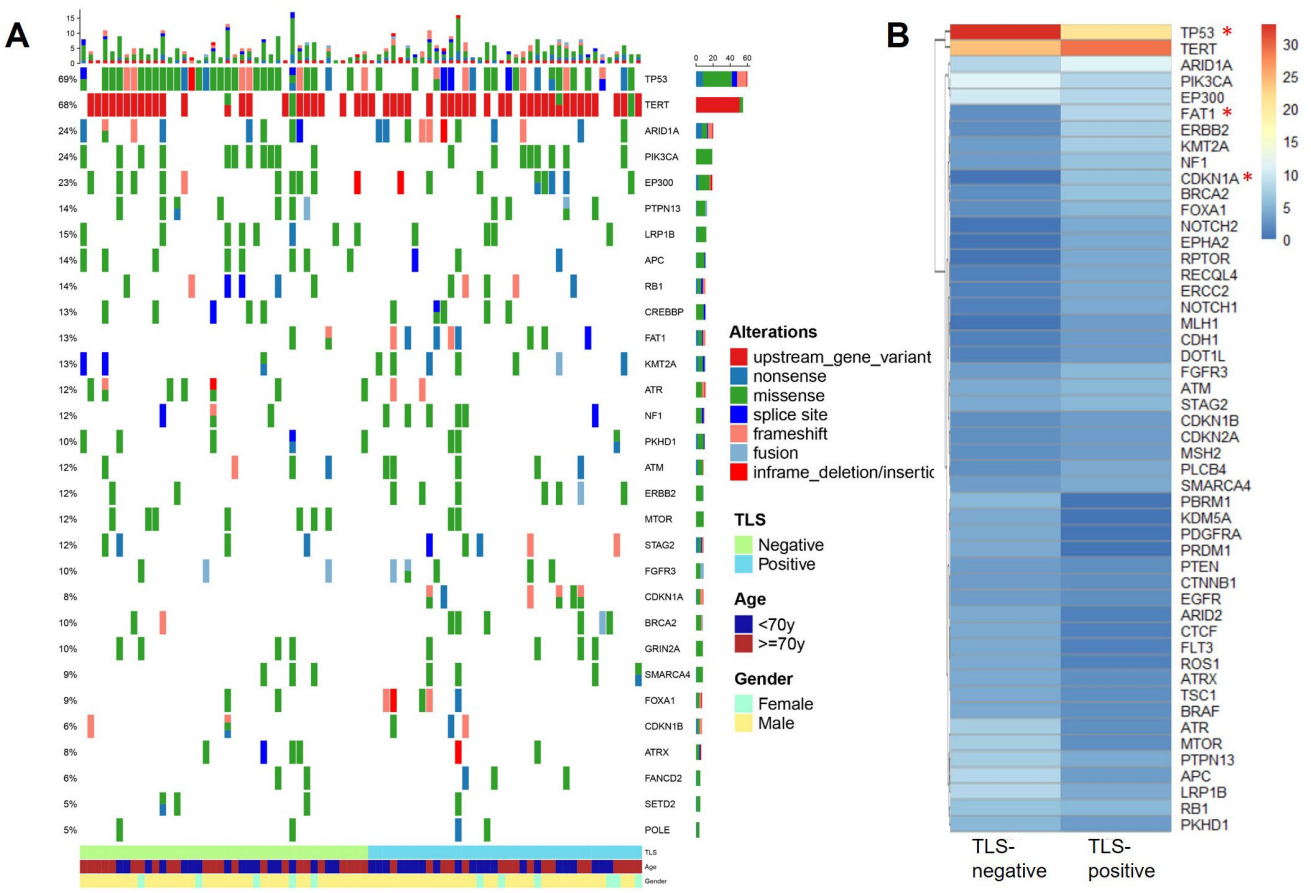
The molecular characteristics of TLS in MIBC. (A) Mutational spectrum of the patients grouped according to TLS status. (B) Heatmap for comparison of genomic alteration frequencies between TLS‐positive and TLS‐negative groups. *, *p* < 0.05.

Within our cohort, 1006 variants across 282 genes were identified (Figure 3A). High TMB (TMB ≥ 10) was observed in 56% (45/80) of cases, with only one case exhibiting MSI, which was positive for TLS. The most frequently mutated genes were *TP53* (68%) and *TERT* (66%), followed by *ARID1A*, *PIK3CA*, and *EP300*. Comparative analysis of the mutation rates among the top 50 genes revealed a lower incidence of *TP53* mutations in TLS‐positive cases compared to TLS‐negative ones (53.8% vs. 80.5%, *p* = 0.011). Additionally, higher mutation rates for *CDKN1A* (15.4% vs. 0%, *p* = 0.011) and *FAT1* (20.5% vs. 4.9%, *p* = 0.045) were observed in TLS‐positive cases. Examination of canonical cancer‐related pathways disclosed no significant TLS‐status dependent differences, except for the cell cycle pathway (Table S4).

### 3.4 *TP53* Mutations Inhibited TLS Production by Inhibiting the Immunogenicity of Neoantigens

**FIGURE 4:**
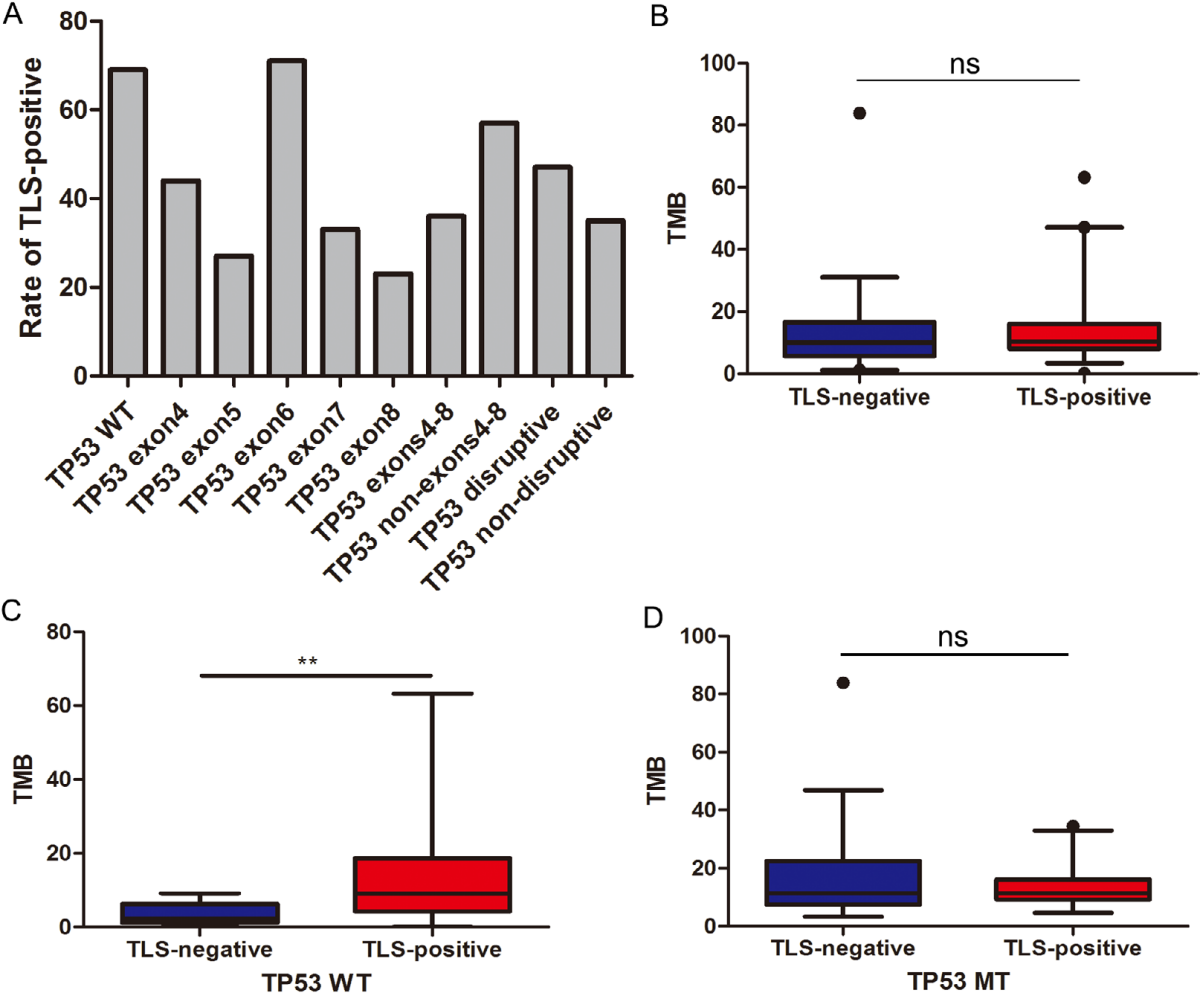
Association between TLS and *TP53* mutations and TMB. (A) The rates of TLS‐positive in cases with *TP53* wild type (WT) status, *TP53* exons4‐8 mutations, *TP53* non‐exons4‐8 mutations, *TP53* disruptive mutations and *TP53* non‐disruptive mutations. Association between TLS status and TMB in all 80 cases (B), *TP53* WT cases (C) and *TP53* mutation type (MT) cases (D). **, *p* < 0.01; ns, *p* > 0.05.

Given the prevalence of *TP53* mutations, we delved deeper into their association with TLS to elucidate why such mutations are associated with a reduction in TLS frequency. *TP53* mutations in our cohort were predominantly located in exons 4–8, which are critical DNA‐binding domains (Figure 4A). This distribution is in line with existing literature [10]. Patients with *TP53* mutations in these exons, excluding exon 6 due to a small sample size, showed a tendency toward fewer TLS. When focusing on disruptive *TP53* mutations—defined as those involving truncating mutations or nonsynonymous alterations in the L2 to L3 loop region [11] —the trends, while present, were less pronounced.

Neoantigens, generated by somatic mutations, are hypothesized to activate immune responses by being presented to effector cells, with their abundance closely tied to TMB [12]. We postulated that a high TMB might trigger TLS formation. However, no significant correlation was observed between TMB and TLS, CD8+ T cells, or B cells within our cohort (Figure 4B, Figure S1). Intriguingly, when *TP53* mutation status was taken into account, TMB showed a significant correlation with TLS in *TP53* wild‐type patients, but not in those with *TP53* mutations (Figure 4C,D, Figure S1). Collectively, these findings suggest that *TP53* mutations may suppress the immunogenic potential of neoantigens, thereby inhibiting TLS formation.

## 4 Discussion

TLSs are acknowledged for their antitumor effects in a wide range of malignancies, including colorectal, breast, and non‐small cell lung cancers [13, 14, 15, 16]. However, in clear cell renal carcinoma, an inverse relationship is observed, where increased TLS density is linked to poorer patient outcomes [7]. Our study adds to this body of evidence by demonstrating that TLSs in MIBC are associated with a lower T‐stage and serve as an independent predictor of favorable prognosis.

The precise mechanisms through which TLS contribute to anti‐tumor processes remain elusive but are likely to involve cellular immunity. Numerous studies have established a link between TLS and the presence of Th1 and CD8+ T cells [8, 17]. In our cohort, although TLS were significantly associated with CD8+ T cell density, the beneficial prognostic impact of TLS was not attributed to CD8+ T cells but rather to B cells. This is consistent with existing literature [18, 19, 20], which suggests that TLS predominantly exert their antitumor effects through B cells. B cells have the potential to impact tumors either by differentiating into plasma cells that produce tumor‐specific antibodies or by acting as antigen‐presenting cells to enhance T cell responses [21]. Plasma cells could be generated in mature TLS but are absent or rare in immature TLS [19, 20]. In our cohort, there were few mature TLS, mostly immature TLS. However, TLS were still significantly associated with plasma cells, suggesting that immature TLS have some mechanism by which plasma cells could be recruited. Similar to CD8+ T cells, the prognostic effect of TLS did not depend on a high density of plasma cells, suggesting that B cells in TLS may primarily play an antigen‐presenting role.

The genesis of TLS is a multifaceted process influenced by various lympho‐organogenic chemokines in response to inflammatory stimuli [22, 23]. The interplay between TLS and genetic mutations within tumor cells is not fully understood. The association between TLS and mutations in the PI3K‐mTOR pathway in renal clear cell carcinoma [7], *BRAF* mutations in colorectal carcinoma [18], and *APC* germline mutations in hepatoblastoma [24] has been reported in studies. Our NGS analysis of 80 MIBC cases revealed that TLS‐positive tumors had lower *TP53* mutation rates and higher *CDKN1A* and *FAT1* mutation rates, suggesting that certain genetic alterations may either promote or inhibit TLS formation.

*TP53* mutations, a common occurrence in human cancers [11, 25], can influence the immune microenvironment in complex ways [26, 27]. While *TP53* mutations are known to elevate TMB [28, 29] and may therefore be expected to enhance TLS formation and immune response, our results indicate that *TP53* mutations may actually suppress TLS formation. This suppression could be due to an inhibitory effect on the immunogenicity of neoantigens derived from TMB. Our findings underscore the need for a deeper understanding of the intricate relationship between *TP53* mutations, TLS, and the immune microenvironment in MIBC.

## 5 Conclusions

Our analysis of TLS in MIBC has identified them as a favorable prognostic factor, whose formation appears to be modulated by *TP53* mutations. Despite these insights, limitations warrant further investigation. Elucidating the specific mechanisms underlying TLS formation will be instrumental in enhancing our comprehensive understanding of the immune microenvironment in MIBC.

## Author Contributions

**Xiaodong Teng:** conceptualization (equal), funding acquisition (equal), project administration (equal), supervision (equal), writing – original draft (equal). **Zhen Chen:** data curation (lead), methodology (equal), validation (equal). **Yanfeng Bai:** data curation (equal), formal analysis (equal), validation (equal). **Hui Cao:** data curation (equal), methodology (equal). **Jing Zhang:** funding acquisition (equal), methodology (equal). **Liming Xu:** methodology (equal). **Kaihua Liu:** methodology (equal), software (equal). **Yuqian Shi:** methodology (equal). **Yang Shao:** methodology (equal).

## Ethics Statement

The present study and an application to waive informed consent were approved by the Ethics Committee of the First Affiliated Hospital, Zhejiang University School of Medicine.

## Consent

The authors have nothing to report.

## Conflicts of Interest

Kaihua Liu, Yuqian Shi, Yang Shao are employees of Nanjing Geneseeq Technology Inc. other authors declare no conflicts of interest.

## Supporting information


**Figure S1.** Association between TMB and tumor‐infiltrating lymphocytes under different TP53 mutation statuses in MIBC. TMB and the densities of CD8+ T cells (A), B cells (B), plasma cells (C) in all cases; TMB and the densities of CD8 + T cells (D), B cells (E), plasma cells (F) in TP53 WT cases; TMB and the densities of CD8 + T cells (G), B cells (H), plasma cells (I) in TP53 MT cases; WT, wild type; MT, mutation type.

**Table S1.** The information of antibodies used in this study.

**Table S2.** The 425 cancer‐related genes included in the GeneseeqPrime panel.

**Table S3.** The correlation between clinicopathological characteristic (or combined factors) and OS in patients with MIBC.

**Table S4.** The percentage of samples with alterations in each of the signaling pathways in which genomic alterations related to MIBC development in terms of TLS status.

## Data Availability

The datasets used and during the current study are available from the corresponding author on reasonable request.
