# Supplementary material for: Tertiary Lymphoid Structures as Independent Predictors of Favorable Prognosis in Muscle‐Invasive Bladder Cancer
Source: Cancer Med. 2025 May 21;14(10):e70978. doi: 10.1002/cam4.70978 (PMC12093152; doi:10.1002/cam4.70978)
Supplement: Supplementary file 1 — Figure S1. Association between TMB and tumor‐infiltrating lymphocytes under different TP53 mutation statuses in MIBC. TMB and the densities of CD8+ T cells (A), B cells (B), plasma cells (C) in all cases; TMB and the densities of CD8 + T cells (D), B cells (E), plasma cells (F) in TP53 WT cases; TMB and the densities of CD8 + T cells (G), B cells (H), plasma cells (I) in TP53 MT cases; WT, wild type; MT, mutation type. [file CAM4-14-e70978-s001.pdf]

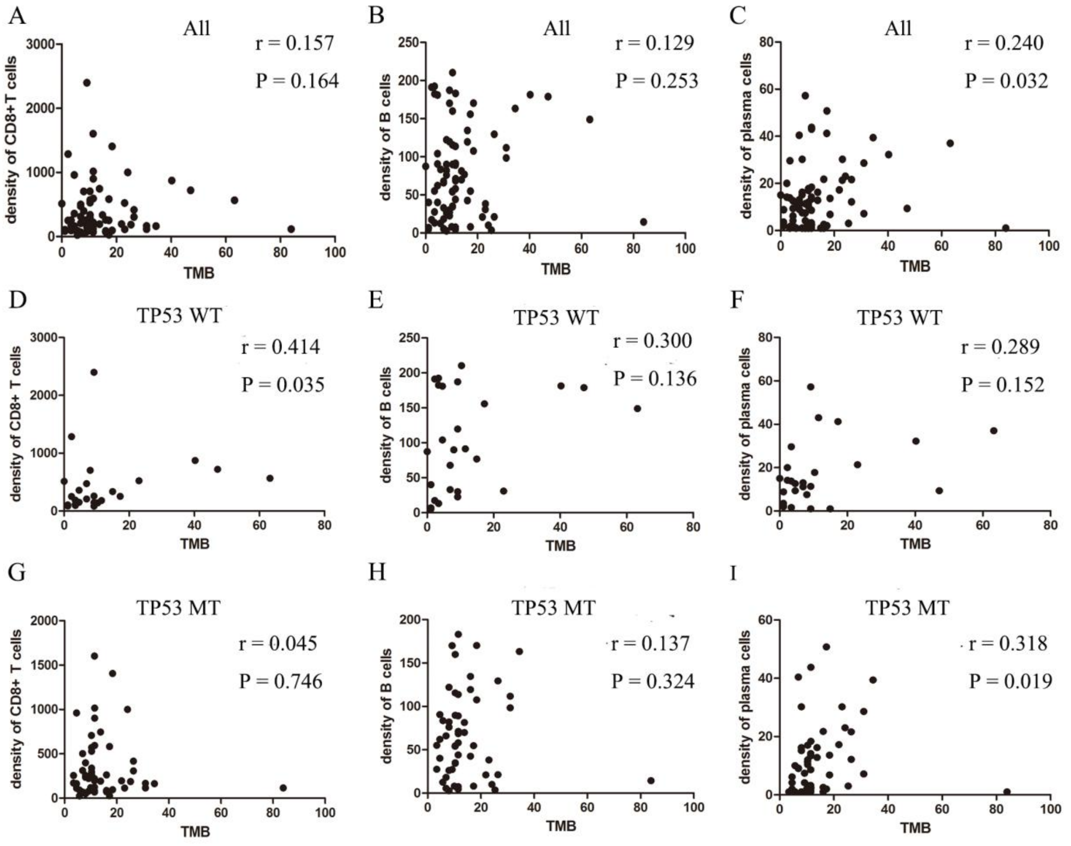

Figure S1. Association between TMB and tumor-infiltrating lymphocytes under different *TP53* mutation statuses in MIBC. TMB and the densities of CD8+T cells (A), B cells (B), plasma cells (C) in all cases; TMB and the densities of CD8+T cells (D), B cells (E), plasma cells (F) in *TP53* WT cases; TMB and the densities of CD8+T cells (G), B cells (H), plasma cells (I) in *TP53* MT cases; WT, wild type; MT, mutation type.
