# Supplementary material for: Tertiary Lymphoid Structures as Independent Predictors of Favorable Prognosis in Muscle‐Invasive Bladder Cancer
Source: Cancer Med. 2025 May 21;14(10):e70978. doi: 10.1002/cam4.70978 (PMC12093152; doi:10.1002/cam4.70978)
Supplement: Supplementary file 2 — Table S1. The information of antibodies used in this study. [file CAM4-14-e70978-s004.docx]

Table S1 The information of antibodies used in this study.

| Antibody | Clone | Company Information |
| --- | --- | --- |
| CD3 | LN10 | Zhongshan Golden Bridge, Beijing, China |
| CD8 | 4B11 | Leica, Germany |
| CD20 | 7D1 | Leica, Germany |
| CD138 | EP201 | Zhongshan Golden Bridge, Beijing, China |
| PD-L1 | SP263 | Ventana |
