# Supplementary material for: Tertiary Lymphoid Structures as Independent Predictors of Favorable Prognosis in Muscle‐Invasive Bladder Cancer
Source: Cancer Med. 2025 May 21;14(10):e70978. doi: 10.1002/cam4.70978 (PMC12093152; doi:10.1002/cam4.70978)
Supplement: Supplementary file 4 — Table S3. The correlation between clinicopathological characteristic (or combined factors) and OS in patients with MIBC. [file CAM4-14-e70978-s002.docx]

Table S3. The correlation between clinicopathological characteristic (or combined factors) and OS in patients with MIBC.

| Factors | OS(m) | *P* |
| --- | --- | --- |
| TLS |  |  |
| Negative | 16 |  |
| Positive | 26 | 0.001 |
| Gender |  |  |
| Female | 26 |  |
| Male | 21 | 0.077 |
| Age |  |  |
| ＜70y | 26 |  |
| ≥70y | 18 | 0.001 |
| Tumour size |  |  |
| ＜3.8cm | 23 |  |
| ≥3.8cm | 21 | 0.64 |
| PD-L1(TPS) |  |  |
| Negative | 21 |  |
| Positive | 24 | 0.346 |
| PD-L1(IPS) |  |  |
| Negative | 21 |  |
| Positive | 27 | 0.068 |
| PD-L1(CPS) |  |  |
| Negative | 20 |  |
| Positive | 24 | 0.137 |
| CD8+T cell density |  |  |
| Low | 19 |  |
| High | 24 | 0.029 |
| Plasma cell density |  |  |
| Low | 19 |  |
| High | 24 | 0.180 |
| B cell density |  |  |
| Low | 20 |  |
| High | 24 | 0.065 |
| Lymph node metastasis |  |  |
| Without | 20 |  |
| With | 18 | 0.516 |
| TNM-stage |  |  |
| II | 28 |  |
| III | 20 |  |
| IV | 20 | 0.084 |
| T-stage |  |  |
| 2 | 27 |  |
| 3 | 20 |  |
| 4 | 19 | 0.055 |
| N-stage |  |  |
| 0 | 22 |  |
| 1 | 16 |  |
| 2 | 21 | 0.408 |
| Vascular invasion |  |  |
| Without | 24 |  |
| With | 19 | 0.089 |
| Nerve invasion |  |  |
| Without | 23 |  |
| With | 16 | 0.009 |
| NLR |  |  |
| Low | 23 |  |
| High | 20 | 0.187 |

MIBC: muscle-invasive bladder cancer; TLS: tertiary lymphoid structure; TPS: tumor proportion score; IPS: immune cell proportion score; CPS: combined positive score; OS: overall survival; NLR: neutrophil-to-lymphocyte ratio.
