# Supplementary material for: Tertiary Lymphoid Structures as Independent Predictors of Favorable Prognosis in Muscle‐Invasive Bladder Cancer
Source: Cancer Med. 2025 May 21;14(10):e70978. doi: 10.1002/cam4.70978 (PMC12093152; doi:10.1002/cam4.70978)
Supplement: Supplementary file 5 — Table S4. The percentage of samples with alterations in each of the signaling pathways in which genomic alterations related to MIBC development in terms of TLS status. [file CAM4-14-e70978-s005.docx]

Table S4. The percentage of samples with alterations in each of the signaling pathways in which genomic alterations related to MIBC development in terms of TLS status.

| Pathway | All | TLS-negative | TLS-positive | P value |
| --- | --- | --- | --- | --- |
| RTK-RAS pathway | 35 of 80 (43.8%) | 17 of 41 (41.5%) | 18 of 39 (46.2%) | 0.673 |
| PI3K pathway | 30 of 80 (37.5%) | 18 of 41 (43.9%) | 12 of 39 (30.8%) | 0.224 |
| Cell cycle pathway | 58 of 80 (72.5%) | 35 of 41 (85.4%) | 23 of 39 (59.0%) | 0.008 |
| Notch signaling pathway | 30 of 80 (37.5%) | 15 of 41 (36.6%) | 15 of 39 (38.5%) | 0.862 |
| TP53 pathway | 58 of 80 (72.5%) | 33 of 41 (80.5%) | 25 of 39 (64.1%) | 0.100 |

MIBC: muscle-invasive bladder cancer; TLS: tertiary lymphoid structure.
